# Non-Contact Measurement of LED Junction Temperature Based on Normalized Integral Width (NIW) of the Emission Spectrum

**DOI:** 10.3390/s26144495

**Published:** 2026-07-15

**Authors:** Fuchun Jiang, Yunming Qiu

**Affiliations:** 1 College of Physics and Optoelectronics Engineering, Shenzhen University, Shenzhen 518060, China; phyopto@163.com; 2 State Key Laboratory Radio Frequency Heterogeneous Integration, Shenzhen 518060, China; 3 Key Laboratory of Optoelectronic Devices and System of Ministry of Education and Guangdong Province, Shenzhen 518060, China; 4 Shenzhen Key Laboratory of Photonic Devices and Sensing Systems for Internet of Things, Shenzhen 518060, China

**Keywords:** LED, junction temperature, non-contact measurement, normalized spectral integral width (NIW), spectral analysis, thermal management, forward voltage method (FVM)

## Abstract

Junction temperature (*T_j_*) is a key parameter that directly governs the optical performance and operational reliability of light-emitting diodes (LEDs), which have become indispensable in modern illumination and display systems. Accurate real-time *T_j_* monitoring is critical for ensuring device longevity and consistent light output. Although the forward voltage method (FVM) remains the industry benchmark, its practical implementation is hindered by the need for costly high-speed switching modules and ultra-low-current calibration sources, restricting its deployment in real-time and cost-sensitive scenarios. To overcome these limitations, we introduce and experimentally validate a non-contact optical method for *T_j_* determination that leverages the normalized integral width (NIW) of the LED emission spectrum as a temperature-sensitive spectral parameter. The underlying principle is that spectral broadening—arising from enhanced carrier thermal excitation and temperature-induced bandgap shrinkage—exhibits a robust and quantifiable linear correlation with *T_j_*. Both theoretical analysis and experimental data confirm that this mechanism underpins the excellent linear correlation between NIW and *T_j_* observed across a wide range of LED types, including monochromatic (red, green, blue) and phosphor-converted white LEDs. A rigorous theoretical analysis establishes the mathematical framework linking NIW to *T_j_*. Experimentally, a measurement system centered on a modified commercial spectrometer was constructed. Extensive testing on a diverse array of power LEDs consistently demonstrates an excellent linear correlation (R^2^ > 0.998) between NIW and *T_j_* under normal drive conditions (e.g., typical operating currents). A comparative analysis against the benchmark FVM, conducted using a Mentor Graphics T3Ster system, demonstrates that the proposed method achieves comparable measurement accuracy, with a maximum deviation of merely 2.1 °C, while substantially reducing system cost and complexity. Validation across diverse LED types confirmed excellent linearity and high repeatability. A comparative analysis with established optical methods (e.g., peak wavelength, blue-white ratio, Raman thermography) further underscores the advantages of the NIW method in terms of cost-effectiveness, measurement speed, and broader applicability. Subsequent evaluation of critical factors, including self-heating, ambient light interference, and spectrometer resolution, demonstrates its robustness. Consequently, the NIW method presents a practical solution for real-time, non-intrusive thermal monitoring, well-suited for industrial LED production and quality control.

## 1. Introduction

Solid-state lighting based on light-emitting diodes (LEDs) has undergone rapid development over the past two decades, fundamentally reshaping the illumination industry. The superior energy efficiency, extended operational lifetime, and environmental friendliness of LEDs have positioned them as the preferred solution for general lighting, display backlighting, and specialized applications, progressively displacing conventional incandescent and fluorescent technologies [1,2].

As LED devices continue to scale toward higher power densities and greater miniaturization, thermal dissipation has emerged as a primary constraint on device performance and reliability [3,4]. Approximately 70% of the input power is dissipated as heat within the tiny PN junction, leading to a significant temperature rise during normal operation. This localized heating elevates the junction temperature ($T_{j}$), which in turn degrades multiple performance metrics—including luminous efficacy, peak wavelength stability, and color temperature consistency—while also accelerating degradation mechanisms that reduce the operational lifetime [5,6,7]. Consequently, $T_{j}$ constitutes a fundamental metric that governs both LED efficiency and thermal behavior. These temperature-dependent effects underscore the critical need for accurate, rapid, and non-invasive $T_{j}$ measurement techniques [8].

Despite extensive research efforts, existing $T_{j}$ measurement techniques face inherent limitations [5,9]. The forward voltage method (FVM), while offering high accuracy, relies on sophisticated and costly instrumentation—including high-speed switching modules and precision low-current sources—which restricts its applicability in real-time or cost-limited settings [10,11,12]. Among non-contact alternatives, the peak wavelength method exploits the temperature-dependent redshift of the electroluminescence peak, but its utility is constrained by weak temperature sensitivity (typically 0.05–0.1 nm/°C for GaN-based devices) and its restriction to narrow-band monochromatic LEDs [13]. The blue-white ratio method is specifically designed for phosphor-converted white LEDs, but it requires separation of the blue and yellow components and is invalid for monochromatic devices [14,15]. Raman spectroscopy and thermoreflectance imaging offer high spatial resolution but have limited accuracy, are expensive, and are impractical for online monitoring [16].

To overcome these limitations, we introduce and experimentally validate a non-contact spectral-based method for $T_{j}$ determination that leverages the normalized integral width (NIW) of the LED emission spectrum as a temperature-sensitive spectral parameter. The proposed NIW-based method offers three key advantages: (i) it utilizes a spectral parameter that is inherently more robust against noise compared to single-point metrics such as peak wavelength, and is applicable across both monochromatic and white LEDs; (ii) it streamlines calibration by employing the normal operating current, thereby eliminating the need for ultra-low-current sources and high-speed switching hardware; and (iii) it is implementable as a complete sensor system using a cost-effective modified commercial spectrometer.

## 2. Theory

The emission spectrum of an LED is governed by the electroluminescence process occurring within the semiconductor active region. Unlike incandescent sources, LED emission is spectrally narrow and contains negligible infrared or ultraviolet components. The dissipated electrical energy is primarily converted into visible light and heat. Due to factors like quantum wells and phosphor materials, non-radiative recombination during light emission generates significant heat accumulated at the PN junction, causing $T_{j}$ to rise. High $T_{j}$, in turn, it intensifies non-radiative thermal activation of electron-hole pairs. The number of non-radiative recombination defects also increases with rising $T_{j}$, leading to reduced light output and a further temperature increase [17].

In GaN-based LEDs—the dominant material system for visible-spectrum emission—the bandgap energy ($E_{g}$) exhibits a strong temperature dependence, decreasing monotonically with increasing temperature. This behavior is quantitatively described by the Varshni relation [Equation (1)], which captures the effect of lattice expansion and electron-phonon interactions on the band structure.

(1) $$E_{g}\left(T\right)=E_{g}\left(0\right)-\frac{\alpha T^{2}}{T+\beta }$$

where α and β are material-specific constants, and $E_{g}(0)$ is the bandgap at 0 K. The corresponding emission wavelength λ is:

(2) $$\lambda =\frac{hc}{E_{g}\left(T\right)}$$

where h is Planck’s constant and c is the speed of light.

The spectral broadening of LED emission with increasing $T_{j}$ originates from three physically distinct but interconnected mechanisms: bandgap renormalization (shifting the peak position), carrier thermal excitation (expanding the energy distribution of occupied states), and energy band broadening and splitting (increasing the intrinsic width of the band structure). While the first mechanism primarily affects the peak wavelength, the latter two are the dominant contributors to the increase in spectral integral width, which is the basis of the NIW method.

First, bandgap renormalization describes the temperature-induced narrowing of the semiconductor bandgap. As described by the Varshni relation in Equation (1), the bandgap energy $E_{g}$ decreases with rising temperature, causing the emission peak to redshift [4,10].

Second, carrier thermal excitation is the primary mechanism contributing to spectral broadening. According to the Boltzmann distribution, the occupation probability of carriers at energy E above the band edge follows $f(E)\propto exp(-E/k_{B}T)$, where $k_{B}$ is the Boltzmann constant. As $T_{j}$ increases, the thermal tail of the carrier distribution extends further into the bandgap, causing carriers to occupy a wider range of energy states. Consequently, electron-hole recombination occurs across a broader energy spectrum, directly increasing the spectral width and thus the NIW value.

Third, energy band broadening and splitting further enhance the spectral width. With rising $T_{j}$, enhanced electron-phonon scattering broadens the energy bands themselves, and intensified collective motion of electrons leads to more pronounced sub-band splitting. Consequently, a broader range of states near the conduction band minimum ($E_{c}$) and valence band maximum ($E_{v}$) contributes to light emission, directly increasing the overall spectral width, which is captured by the NIW.

**Figure 1:**
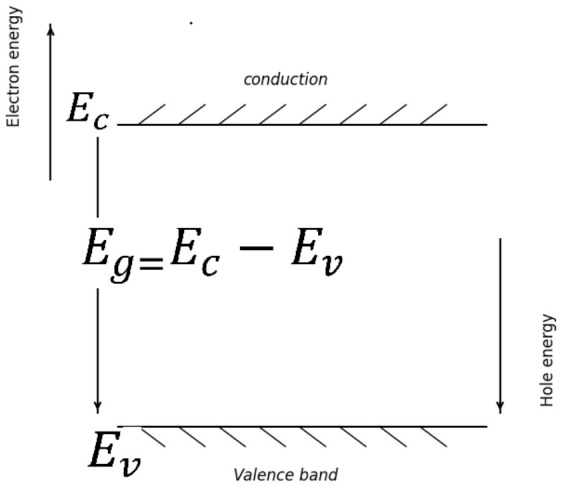
Schematic illustration of temperature-induced spectral broadening in LED emission, arising from bandgap renormalization, carrier thermal excitation, and energy band broadening and splitting. These three mechanisms are collectively captured by the normalized integral width (NIW).

In summary, among the three temperature-dependent mechanisms, carrier thermal excitation and energy band broadening and splitting are the dominant processes that directly broaden the emission spectrum and, consequently, increase the normalized integral width. The NIW method, by integrating over the full spectral power distribution (380–780 nm), inherently captures these broadening effects, forming the physical foundation for its application in junction temperature measurement. In contrast, single-point spectral parameters such as peak wavelength cannot fully capture these combined effects. This comprehensive sensitivity to full-spectrum broadening, as illustrated in Figure 1 below, is the key advantage of the NIW method over conventional spectral parameters.

It should be noted that while the microscopic mechanisms underlying spectral broadening—such as bandgap renormalization and carrier thermal excitation—may vary across different LED structures (e.g., single junction, double heterojunction, or multi-quantum-wells), the NIW method operates at the macroscopic device level. The junction temperature $T_{j}$ is a well-defined macroscopic parameter representing the average temperature of the PN junction region, and the spectral broadening measured by NIW consistently correlates with $T_{j}$ regardless of the specific internal structure. This is confirmed by our experimental results across multiple LED types with different structures.

## 3. Measurement Principle

The emission spectrum of an LED is sensitive to both the drive current and the junction temperature. As $T_{j}$ varies, the spectral profile undergoes systematic changes, including broadening and peak shifting. To quantify the spectral broadening effect, we define a dimensionless metric—the normalized integral width (NIW)—which measures the spread of the emission spectrum. Mathematically, NIW is uniformly defined as the integral width between the two valley points (local minima) adjacent to the dominant emission peak, normalized by the peak intensity:

(3) $$\mathrm{NIW}=\frac{\int_{{\lambda v}_{1}}^{{\lambda v}_{2}}P\left(\lambda \right)d\lambda }{P_{peak}}$$

where P(λ) is the normalized spectral intensity distribution, $P_{\mathrm{peak}}$ is the normalized peak intensity of the dominant emission band, and the integration limits $\lambda_{v1}$ and $\lambda_{v2}$ are adaptively determined by the two valley points adjacent to the dominant peak.

The determination of the integration interval depends on the LED type: (i) For monochromatic LEDs (red, green, blue), the spectrum consists of a single isolated emission band. The two valley points are well-defined at the baseline boundaries of the spectrum. For practical implementation, the integration limits are set to $\lambda_{v1}=380$ nm and $\lambda_{v2}=780$ nm, which consistently encompass the entire emission band across different monochromatic LEDs. (ii) For YAG-type phosphor-converted white LEDs, the emission spectrum comprises a blue peak from the InGaN chip and a broad yellow emission band from the YAG:Ce^3+^ phosphor. In this work, the NIW is computed exclusively on the blue emission peak, with the integration window ($\lambda_{v1},\lambda_{v2}$) adaptively defined by the two valley points surrounding the blue peak: one on the short-wavelength side of the blue peak, and the other at the local minimum between the blue peak and the yellow phosphor band. This adaptive definition ensures two essential properties: ① the NIW consistently captures the temperature-induced broadening of the blue chip emission without being influenced by the phosphor-related yellow component, which is excluded from the NIW calculation for white LEDs; and ② the integration window automatically tracks any temperature- or aging-induced shifts in the blue peak, rather than relying on a fixed wavelength window.

The variation of NIW with junction temperature can be expressed as:

(4) $$NIW=f(T_{j},I)$$

where the function f describes the temperature-dependent spectral response of the LED, which is characterized by the NIW-$T_{j}$ coefficient. This coefficient varies with operating current (I). Under a fixed operating current I, the relationship between NIW and $T_{j}$ can be linearized as:

(5) $$\mathrm{NIW}=k\cdot \Delta T_{j}+{\mathrm{NIW}}_{0}$$

where $\Delta T_{j}=T_{j}-T_{0}$, $T_{j}$ denoting the junction temperature under the target normal drive conditions, $T_{0}$ reference the junction temperature (both at the same drive current); ${NIW}_{0}$ and NIW are the corresponding NIW values at $T_{0}$ and $T_{j}$, respectively. The coefficient *k* (in %/°C) represents the sensitivity of NIW to $T_{j}$, and is established through experimental calibration. Thus, once k, $T_{0}$, and the ${NIW}_{0}$ are determined, $T_{j}$ under arbitrary operating conditions can be determined from the measured NIW using Equation (6).

(6) $$T_{j}=\frac{NIW-{NIW}_{0}}{k}+T_{0}$$

Unlike conventional spectral metrics such as the full width at half maximum (FWHM)—which rely on a single data point—the NIW offers superior robustness against random noise by utilizing the entire profile of the dominant emission peak. This property makes NIW particularly advantageous in industrial environments where measurement noise is prevalent.

## 4. Experiment

### 4.1. Test Samples

To ensure broad applicability of the measurement method, commercial power LEDs of four different emission colors (red, green, blue, and phosphor-converted white) were acquired from standard production lots. Prior to thermal characterization, a thin and uniform layer of thermal grease was applied to the base plates of each LED to minimize thermal contact resistance [13].

### 4.2. Experimental Setup

The junction temperature measurement system based on normalized integral width consists of:

1. An ATA-500 spectrometer (EVERFINE Corporation, Hangzhou, China; spectral range 380–780 nm, resolution 0.5 nm, wavelength accuracy ±0.2 nm, integration time adjustable from 1 ms to 9 ms, signal-to-noise ratio 1000:1 at full signal)
2. A constant temperature chamber (CL-200, EVERFINE Corporation, Hangzhou, China; stability ±0.1 °C)
3. An integrating sphere (EVERFINE Corporation, Hangzhou, China; 50 cm diameter, BaSO_4_-coated inner wall, 2 cm port for optical fiber);
4. A high-precision constant current source (Keithley 2450, Keithley Instruments, Cleveland, OH, USA; accuracy ±0.02%)
5. A set of mounting fixtures and a light-tight sealed enclosure to eliminate ambient light.

**Figure 2:**
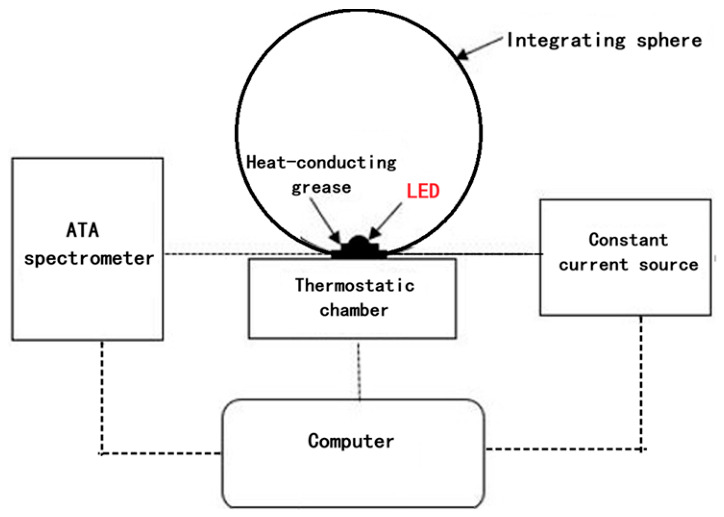
Experimental setup for NIW-based junction temperature measurement.

A fiber-optic probe was coupled to the spectrometer input, with its detection end positioned at the integrating sphere’s sampling port to capture the LED emission. The setup is illustrated in Figure 2.

The raw spectral data acquired from the spectrometer are first background-subtracted using a dark spectrum measured with the LED off. The resulting spectrum is then normalized to its peak intensity. Subsequently, the NIW is numerically integrated over the integration window defined in Section 3—i.e., between the two valley points adjacent to the dominant emission peak—using the trapezoidal rule.

### 4.3. Measurement Procedure

Throughout this study, the normal operating current refers to the rated drive current of the LED under test (e.g., 350 mA for the 1 W power LEDs used here), which is significantly higher than the ultra-low calibration current (typically ≤1 mA) employed in the FVM. The NIW method performs calibration directly at this normal operating current, thereby eliminating the need for current switching between calibration and measurement modes.

The procedure comprised three stages: data acquisition, calibration, and junction temperature measurement.

(1) Data Acquisition:
  - The LED (with thermal grease) was firmly attached to the temperature probe inside the chamber to ensure good thermal contact.
  - The chamber was set to an initial temperature $T_{1}$. The system was stabilized for about 30 min to achieve thermal equilibrium among the substrate, chip, and chamber.
  - The LED was driven at its normal operating current (e.g., the rated current specified by the manufacturer). The modified measurement system was triggered to rapidly acquire the emission spectrum, and the normalized integral width ${NIW}_{1}$ was computed.
  - The chamber temperature was increased in equal temperature increments. Step (c) was repeated at each temperature setpoint to obtain the corresponding integral width ${NIW}_{i}$.
(2) Calibration:

A key distinction of the NIW method relative to the FVM is that calibration is performed at the normal operating current (e.g., the rated current), rather than at a very low current. This eliminates the need for ultra-low-current sources and associated calibration procedures. While self-heating does occur under the normal drive current, the junction temperature rise remains effectively constant for a given current within the short duration (milliseconds) of spectral acquisition. To minimize any residual self-heating effects, the room-temperature state is adopted as the reference condition, with $T_{0}$ and ${NIW}_{0}$ denoting the reference temperature and corresponding NIW value, respectively.

The reference state is established at room temperature, denoted by $T_{0}$ and the corresponding ${NIW}_{0}$. The differences in NIW and ${NIW}_{j}$ under other different conditions, item by item as follows:

(7) $$\Delta \mathrm{NIW}={\mathrm{NIW}}_{i}-{\mathrm{NIW}}_{0}$$

(8) $$\Delta T=T_{i}-T_{0}$$

A linear fit is then performed to obtain the calibration function:

(9) ΔNIW=k·ΔT

A plot of ΔNIW versus ΔT is generated, and linear regression yields the calibration function given in Equation (9).

(3) Measurement:

The measurement phase aims to determine the junction temperature of interest $T_{jx}$ under the target operating conditions, following the steps below:

The goal is to determine the actual operating junction temperature $T_{jx}$ under any normal working condition. The steps are:

- Record the emission spectrum under the target operating conditions and calculate the NIW value.
- Determine the NIW change relative to the reference: ΔNIW = NIW −NIW_0_.
- Compute the junction temperature using the calibration function:

(10) $$T_{j}=T_{0}+\frac{\Delta NIW}{k}$$

## 5. Results and Discussion

### 5.1. Experimental Results

#### 5.1.1. Monochromatic LEDs

The normalized spectral intensity distributions of each monochromatic LED were measured at a series of temperature setpoints with equal increments. The NIW was calculated according to the definition in Section 3, with the integration window determined by the valley points adjacent to the dominant emission peak (for monochromatic LEDs, the two valley points correspond to the baseline boundaries of the spectrum). The results are summarized in Table 1.

**Figure 3:**
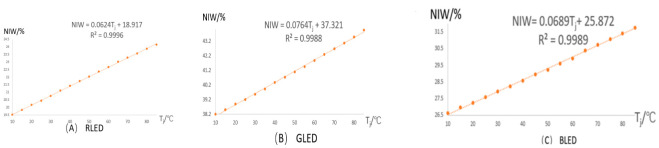
NIW versus junction temperature for monochromatic LEDs (**A**) Red; (**B**) Green; (**C**) Blue.

The NIWs are plotted against junction temperature in Figure 3.

The NIW exhibits an excellent linear correlation with $T_{j}$ for all three monochromatic LEDs under normal operating current. Linear regression of the calibration data yields the following functions:

- Red LED: NIW = 0.0624 $T_{j}$ + 18.917, R^2^ = 0.9996
- Green LED: NIW = 0.0764 $T_{j}$ + 37.321, R^2^ = 0.9988
- Blue LED: NIW = 0.0689 $T_{j}$ + 25.872, R^2^ = 0.9989

All $R^{2}$ values exceed 0.99, confirming excellent linearity. To minimize potential bias from self-heating at the normal operating current and minor chamber temperature fluctuations, room temperature was adopted as the reference state. The corresponding relative integral widths ΔNIW are presented in Table 2.

**Figure 4:**
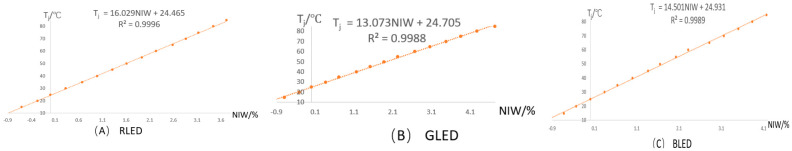
Calibration curves for monochromatic LEDs: (**A**) Red; (**B**) Green; (**C**) Blue.

Calibration curves derived from Table 2 are shown in Figure 4. The fitted functions are:

- Red LED: $\Delta T_{j}$ = 16.029 ΔNIW, R^2^ = 0.9996
- Green LED: $\Delta T_{j}$ = 13.073 ΔNIW, R^2^ = 0.9988
- Blue LED: $\Delta T_{j}$ = 14.501 ΔNIW, R^2^ = 0.9989

The near-perfect linearity (R^2^ > 0.99) confirms the feasibility of using NIW for $T_{j}$ measurement.

#### 5.1.2. White LEDs

**Figure 5:**
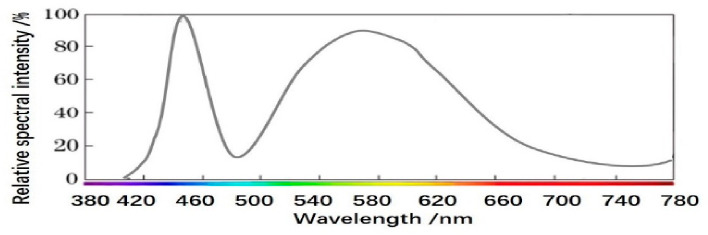
Emission spectrum of a phosphor-converted white.

Commercial white LEDs typically use a blue LED chip to excite YAG:Ce^3+^ phosphor, producing a broad spectrum consisting of blue and yellow emission bands, as shown in Figure 5.

For white LEDs, the spectral valley between the blue peak and the yellow phosphor band was used to determine the right-side boundary of the integration window. The NIW was computed exclusively on the blue emission peak, with the integration window adaptively defined by the two valley points surrounding the blue peak (as described in Section 3), rather than using a fixed wavelength range. Room temperature was adopted as the reference state. The corresponding equivalent normalized integral width (${\mathrm{NIW}}_{eq}$) values are listed in Table 3.

**Figure 6:**
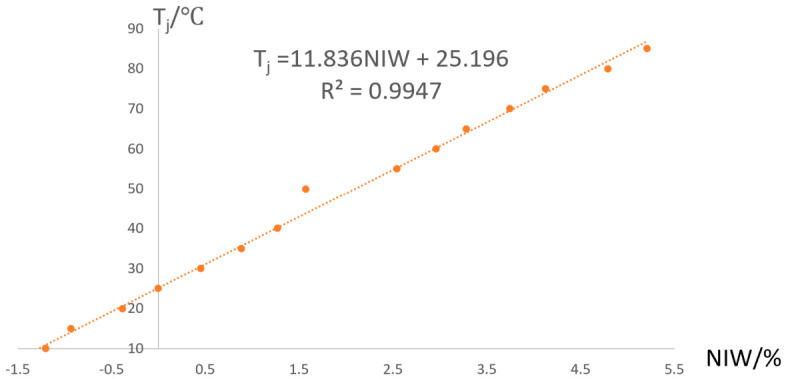
Calibration curve for white LED.

The calibration curve is shown in Figure 6. Linear fitting of the data yielded the calibration function: $\Delta {\mathrm{NIW}}_{eq}$ = 0.0844 ΔT (R^2^ = 0.9947). The NIW-$T_{j}$ coefficient k for this white LED was determined to be 11.836%/°C.

The calibration results for both monochromatic and white LEDs validate that the normalized spectral integral width maintains a strong linear correlation with junction temperature across different LED types. With the established calibration functions, the junction temperature under arbitrary operating conditions can be directly determined from the measured NIW value.

#### 5.1.3. Repeatability Assessment

To evaluate the repeatability of the NIW method, each measurement was repeated five times under identical conditions for each LED type. The standard deviation of NIW values across the five measurements was less than 0.1% for all LEDs, corresponding to a temperature repeatability of ±0.3 °C. This confirms that the NIW method yields consistent and reproducible results, which is essential for industrial quality control.

#### 5.1.4. Influence of Driving Current on NIW Calibration

To evaluate the effect of driving current on the NIW-$T_{j}$ calibration relationship, measurements were performed at multiple current levels (100 mA, 200 mA, 350 mA, and 500 mA) for the four LED types. At each current level, the NIW-$T_{j}$ calibration data were obtained following the same procedure described in Section 4.3, and the NIW-$T_{j}$ calibration slope coefficient k was determined by linear regression. The results are summarized in Table 4.

The NIW-$T_{j}$ linear relationship is well-maintained across the 100–500 mA range for all four LED types, with R^2^ values above 0.99 in all cases. The slope coefficient k exhibits only slight variation with driving current, remaining nearly constant across the tested current range. This indicates that calibration performed at the normal operating current (350 mA) can be applied to other current levels within the 100–500 mA range with acceptable accuracy. For practical applications, recalibration is recommended only when the operating current deviates substantially from the calibration current (e.g., beyond the 100–500 mA range), as larger current changes may introduce more significant variations in the spectral characteristics.

#### 5.1.5. Effect of Long-Term Aging and Phosphor Degradation on NIW Characteristics

LED aging is known to induce gradual degradation of device performance, which can alter the emission spectrum over time. The aging mechanisms differ between monochromatic and phosphor-converted white LEDs, and thus their potential impacts on NIW measurement are also distinct.

For monochromatic LEDs, aging primarily results from degradation of the chip’s internal quantum efficiency, manifested as an increase in non-radiative recombination centers and a reduction in overall emission intensity [18,19]. However, since NIW is a normalized parameter that integrates the relative spectral power distribution rather than absolute intensity, degradation of the internal quantum efficiency alone is expected to have a minimal effect on the NIW-$T_{j}$ slope coefficient k. The spectral shape remains largely unchanged during aging, as reported in previous studies [20]. Therefore, for monochromatic LEDs, the aging effect on NIW measurement accuracy is considered negligible.

For phosphor-converted white LEDs, the NIW is defined as the normalized integral width of the blue emission peak, with integration boundaries adaptively determined by the two inflection points of the blue peak profile (see Section 5.1.2). Since the yellow emission from the phosphor is not included in the NIW integration, phosphor degradation does not affect the NIW directly through the blue-to-yellow ratio. Instead, two distinct aging mechanisms can influence the measured blue peak profile:

(i) Chip degradation—similar to monochromatic LEDs, degradation of the blue chip’s internal quantum efficiency reduces emission intensity but has minimal effect on the normalized blue peak shape.
(ii) Phosphor layer degradation —during prolonged operation, the phosphor/silicone composite layer may undergo structural degradation, including microcracks, delamination, and surface roughening of phosphor particles [21,22]. These effects alter the optical path of the blue light as it passes through the phosphor layer, increasing scattering and re-absorption [21]. As a result, the profile of the blue emission peak—including its symmetry, width, and tailing characteristics—can be modified. Since the NIW integration boundaries are adaptively determined by the actual blue peak shape, such modifications are captured by the NIW value. In addition, thermal degradation of the silicone matrix may lead to changes in refractive index and transmittance, further affecting the measured blue peak profile [23].

Therefore, for white LEDs, the NIW is primarily sensitive to the blue-chip temperature (through bandgap narrowing and carrier thermal broadening) but can also be influenced by phosphor-layer-induced modifications of the blue peak profile during prolonged aging.

Compensation strategy: To mitigate potential aging-induced drift in practical applications, we recommend: (i) periodic recalibration—performing recalibration at the normal operating current at regular intervals (e.g., every 500–1000 h of operation) for both monochromatic and white LEDs; and (ii) blue peak shape monitoring—for white LEDs, since the NIW is adaptively defined by the blue peak profile, tracking its evolution over time provides a direct indicator of aging effects. Recalibration can be triggered when the NIW at a reference temperature deviates beyond a preset threshold. These strategies are expected to effectively mitigate aging-induced drift and maintain reliable measurement accuracy for practical applications.

#### 5.1.6. Summary of Compensation Strategies

Based on the analyses in Section 5.1.4 and Section 5.1.5, the compensation strategies for practical applications can be summarized as follows.

For driving current variation, calibration should be performed at the specific operating current of the target device. A single calibration at the normal operating current can be applied to other current levels within the 100–500 mA range, as the NIW-$T_{j}$ linearity remains excellent across this range. Re-calibration is recommended when the operating current changes substantially (e.g., beyond the 100–500 mA range).

For long-term aging, the impact on monochromatic LEDs is minimal, as the normalized spectral shape remains largely unchanged during aging. Periodic recalibration at regular intervals (e.g., every 500–1000 h) is sufficient to maintain accuracy. For phosphor-converted white LEDs, the primary concern is phosphor-layer degradation, which may modify the blue emission peak profile through increased scattering and re-absorption. In addition to periodic recalibration, blue peak shape monitoring is recommended; recalibration can be triggered when the NIW at a reference temperature deviates beyond a preset threshold.

These strategies are designed to be practical and easily implementable without requiring significant hardware modifications to the measurement system.

### 5.2. Error Analysis and Uncertainty Budget

Several factors contribute to the measurement uncertainty of the NIW method. The estimated error sources and their contributions are summarized in Table 5.

### 5.3. Validation Against the Forward Voltage Method (FVM)

The forward voltage method (FVM) is widely recognized as the benchmark technique for junction temperature measurement [7]. In practice, however, its implementation requires calibration at very low currents (typically 1 mA or lower), which demands high-sensitivity data acquisition systems. In addition, the measurement procedure involves high-speed switching between the operating current and the low calibration current, necessitating specialized and costly hardware modules. The NIW method, by contrast, only requires modest modifications to a standard spectrometer and thus offers a clear cost advantage [9,12].

To verify the accuracy of the NIW method, the monochromatic (red, green, blue) and phosphor-converted white LEDs were measured under identical drive conditions at the same operating current. The junction temperatures obtained from the NIW method were then directly compared with the reference values provided by a commercial T3Ster system (Mentor Graphics, USA), which implements the standard forward voltage technique. The complete comparison is given in Table 6.

For the monochromatic and white LEDs tested, the maximum deviation between the NIW method and the FVM reference is only 2.1 °C, confirming the reliability of the proposed approach. Importantly, this level of accuracy can be obtained without relying on expensive high-speed switching electronics or ultra-low-current calibration sources.

### 5.4. Quantitative Comparison with Peak Wavelength and Blue-White Ratio Methods

To quantitatively evaluate the advantages of the NIW method, parallel comparisons were conducted under identical conditions (350 mA, 10–85 °C) using the peak wavelength method (for monochromatic LEDs) and the blue-white ratio method (for white LEDs). All results were benchmarked against FVM (T3Ster).

For monochromatic LEDs, as summarized in Table 7, the peak wavelength method yielded average errors of 5.1–8.5 °C (maximum 7.6–14.8 °C), significantly higher than those of the NIW method (average <1.2 °C, maximum <2.0 °C).

For white LEDs, the blue-white ratio method showed an average error of 2.5 °C (maximum 4.1 °C), while the NIW method achieved better accuracy (average 1.2 °C, maximum 2.1 °C) with universal applicability to both monochromatic and white LEDs (Table 8).

Anti-interference tests with 5% spectral noise further confirm its robustness: the NIW method maintained error below 1.9 °C, while the peak wavelength and blue-white ratio methods showed errors exceeding 7.6 °C under the same noisy conditions. This superior performance stems from the integral nature of NIW, which averages out random fluctuations in the spectral data.

### 5.5. Comprehensive Comparison with Other Optical Methods

To further highlight the advantages of the NIW method, Table 9 provides a multidimensional comparison with existing optical techniques for junction temperature measurement.

The NIW method offers the best combination of low cost, non-contact operation, fast measurement speed, reasonable accuracy, and wide applicability across different LED types. Unlike the peak wavelength method, NIW works for white LEDs; unlike the blue-white ratio method, it works for monochromatic LEDs. This universality is a key advantage for manufacturing environments where multiple LED types are produced on the same line.

### 5.6. Influence of Spectrometer Resolution

To investigate the effect of spectrometer resolution on measurement accuracy, we repeated the NIW measurements for the red LED using different resolution settings (0.5 nm, 1 nm, 2 nm). The results show that the NIW values change by less than 0.2% when the resolution is varied from 0.5 nm to 1 nm; the NIW changes by less than 0.1%, corresponding to a temperature difference of approximately 0.3 °C. At 2 nm resolution, the error increases to 1.2 °C due to excessive smoothing of the spectral features. Therefore, a spectrometer with a resolution of 2 nm or better is recommended for practical implementations. Most commercial compact spectrometers meet this requirement.

### 5.7. Summary of Advantages

The NIW method offers significant advantages over existing techniques:

(1) Instrumentation simplicity: The method relies on a standard spectrometer with minimal hardware modification, avoiding the costly switching electronics and precision low-current sources that are indispensable for FVM-based measurements. This translates directly into reduced capital investment and lower maintenance overhead.
(2) Measurement transparency: Because the sensing mechanism is purely optical, the method does not interfere with the electrical circuit of the device under test. Continuous real-time monitoring can thus be performed without perturbing the LED’s normal operating state—a feature that is particularly valuable in production environments where uninterrupted operation is required.
(3) Versatility across LED types: The method has been validated on both monochromatic (red, green, blue) and phosphor-converted white LEDs. Its underlying principle—that spectral broadening tracks junction temperature—is generic and can, in principle, be extended to other semiconductor lighting technologies.
(4) Streamlined calibration: Unlike FVM, which requires calibration at very low currents and subsequent high-speed switching, the NIW method performs calibration directly at the normal operating current. This not only simplifies the procedure but also eliminates a potential source of measurement artifacts associated with current transients.
(5) Measurement robustness: By integrating over the entire spectral profile rather than extracting a single point, the NIW is intrinsically less sensitive to random noise and spectral fluctuations, ensuring reproducible results across repeated measurements.

A few caveats should be mentioned. The achievable accuracy is tied to the resolution and stability of the spectrometer employed; routine recalibration may be necessary to compensate for long-term drift. Additionally, one-time calibration per LED type or batch is required, and care must be taken to avoid ambient light contamination—for example, by using an integrating sphere or a light-tight enclosure. The behavior of the method under extreme drive conditions or rapidly changing temperatures warrants further investigation. The current validation covers the 10–85 °C range, which is the typical operating range for commercial power LEDs. While the NIW method is expected to remain effective beyond this range based on its physical mechanism, validation under more extreme temperatures is reserved for future work. At very low temperatures (below 0 °C), reduced carrier injection efficiency may require longer integration times to maintain measurement accuracy. At very high temperatures (above 85 °C), phosphor thermal quenching in white LEDs may introduce additional spectral reshaping effects, which could affect the NIW measurement. Despite these considerations, the overall balance of simplicity, cost, and performance positions the NIW method as a viable alternative to more complex established techniques.

## 6. Conclusions

This work presents a non-contact spectral method for LED junction temperature ($T_{j}$) measurement based on the normalized integral width of the emission spectrum. The method captures temperature-dependent spectral broadening—arising from bandgap shrinkage and carrier thermal redistribution—through the full-spectrum integral width.

Experiments on red, green, blue, and white power LEDs confirm a strong linear correlation between NIW and $T_{j}$ (R^2^ > 0.99). When benchmarked against the FVM using a T3Ster system, the NIW method achieves a maximum deviation of only 2.1 °C, with a repeatability of ±0.3 °C and an overall uncertainty of ±0.64 °C.

Compared to peak-wavelength and blue-white ratio methods, the NIW approach provides a more favorable combination of attributes: it is non-invasive, cost-effective, rapid, and universally applicable to both monochromatic and white LEDs without requiring any method-specific adjustments.

Future efforts will be directed toward enhancing the temporal resolution for dynamic $T_{j}$ monitoring and thermal resistance characterization, extending validation to emerging devices including COB, mini-LED, and micro-LED, and developing a miniaturized spectral sensor for embedded thermal management applications.

## Figures and Tables

**Table 1:** NIW vs. $T_{j}$ for monochromatic LEDs.

$T_{j}$ (°C)	R-LED NIW (%)	G-LED NIW (%)	B-LED NIW (%)
10	19.5290	38.2099	26.6465
15	19.8320	38.5118	26.9635
20	20.1676	38.8921	27.2552
25	20.4418	39.2066	27.5873
30	20.7607	39.5725	27.9280
35	21.1142	39.9177	28.2283
40	21.4319	40.3615	28.5808
45	21.7484	40.7274	28.9505
50	22.0529	41.0772	29.2357
55	22.3738	41.4352	29.6107
60	22.6749	41.8732	29.9015
65	23.0257	42.2661	30.4038
75	23.5649	43.0607	31.0861
80	23.8832	43.4719	31.4192
85	24.1625	43.9426	31.7544

**Table 2:** ΔNIW of Monochromatic LEDs vs. Temperature at 350 mA (Reference: 25 °C).

$T_{j}$ (°C)	R-LED ΔNIW (%)	G-LED ΔNIW (%)	B-LED ΔNIW (%)
10	−0.9127	−0.9966	−0.9408
15	−0.609	−0.694	−0.623
20	−0.2741	−0.3144	−0.3321
25	0.0000	0.0000	0.0000
30	0.3189	0.3659	0.3189
35	0.6724	0.7111	0.6724
40	0.9901	1.1549	0.9901
45	1.3066	1.5208	1.3066
50	1.6111	1.8706	1.6483
55	1.9320	2.2286	2.0233
60	2.2331	2.6666	2.3141
65	2.5839	3.0595	2.8164
75	3.4414	4.2653	3.8318
80	3.7207	4.7360	4.1671
85	3.9272	5.1189	4.4731

**Table 3:** ${\mathrm{NIW}}_{eq}$ as a function of temperature for the white LED at the reference state.

$T_{j}$ (°C)	5	10	15	20
${\mathrm{NIW}}_{eq}$ (%)	−1.5658	−1.2024	−0.9294	−0.3816
$T_{j}$ (°C)	25	30	35	40
${\mathrm{NIW}}_{eq}$ (%)	0.00000	0.4533	0.8854	1.2718
$T_{j}$ (°C)	50	55	60	65
${\mathrm{NIW}}_{eq}$ (%)	1.5698	2.5452	2.9603	3.2831
$T_{j}$ (°C)	70	75	80	85
${\mathrm{NIW}}_{eq}$ (%)	3.750	4.1282	4.7936	5.2093

**Table 4:** NIW-$T_{j}$ calibration slope coefficient k (in %/°C) at different driving currents.

LED Type	Driving Current (mA)			
	100	200	350	500
R-LED	0.0618	0.0621	0.0624	0.0630
G-LED	0.0756	0.0760	0.0764	0.0772
B-LED	0.0682	0.0686	0.0689	0.0696
W-LED	0.0837	0.0841	0.0844	0.0852

*All R^2^ values are above 0.99.*

**Table 5:** Error budget for the NIW method.

Error Source	Contribution (°C)	Mitigation Strategy
Spectrometer resolution (0.5 nm)	±0.2	Use high-resolution spectrometer (0.5 nm or better)
Spectrometer wavelength accuracy (±0.2 nm)	±0.15	Regular calibration with Hg-Ar lamp
Temperature chamber stability (±0.1 °C)	±0.3	Longer stabilization time (30 min)
Self-heating during calibration	±0.5	Short acquisition time (5 ms); reference at 25 °C
Ambient light interference	±0.1	Dark enclosure and background subtraction
NIW computation (numerical integration)	±0.05	Trapezoidal rule with fine wavelength steps
Total root-sum-square (RSS) uncertainty	±0.64 °C	—

**Table 6:** Comparison of junction temperatures determined by the NIW method and the forward voltage method.

LED Type	K ($T_{j}$-NIW Coeff.) (°C/%)	Calib. Lin. (R^2^)	$T_{j}$ by NIW (°C)	$T_{j}$ by FVM (°C)	Difference (°C)
R-LED	16.029	0.9996	49.4	51.2	−1.8
G-LED	13.073	0.9998	57.2	55.3	+1.9
B-LED	14.501	0.9989	68.8	70.4	−1.6
W-LED	11.836	0.9947	73.5	75.6	−2.1

**Table 7:** Comparison of measurement errors: NIW method vs. peak wavelength method (monochromatic LEDs).

LED Type	Method	Average Error (°C)	Maximum Error (°C)
R-LED	Peak wavelength	5.1	7.6
	NIW	0.9	1.8
G-LED	Peak wavelength	6.7	14.5
	NIW	1.1	1.9
B-LED	Peak wavelength	8.5	14.8
	NIW	1.0	1.6

**Table 8:** Comparison of measurement errors: NIW method vs. blue-white ratio method (white LEDs).

Method	Average Error (°C)	Maximum Error (°C)	Applicable LED Types
Blue-white ratio	2.5	4.1	White only
NIW	1.2	2.1	All types

**Table 9:** Comparison of NIW method with other LED junction temperature measurement techniques.

Method	Contact/Non-Contact	Cost	Speed	Applicable LED Types	Instrumentation Required
Forward voltage (FVM)	Contact	High	Slow (needs settling)	All	High-speed switch, low-current source
Peak wavelength	Non-contact	Medium	Fast	Monochrome only	Spectrometer
Blue-white ratio	Non-contact	Low	Fast	White LEDs only	Spectrometer
Raman spectroscopy	Non-contact	Very high	Slow	Lab only	Raman microscope
Thermoreflectance	Non-contact	High	Medium	Bare die only	CCD and lock-in amplifier
NIW (this work)	Non-contact	Low	Fast	All	Modified spectrometer

## Data Availability

The original contributions presented in this study are included in the article. Further inquiries can be directed to the corresponding author.
